# An open future for ecological and evolutionary data?

**DOI:** 10.1186/1472-6785-14-10

**Published:** 2014-04-02

**Authors:** Amye Kenall, Simon Harold, Christopher Foote

**Affiliations:** 1 BioMed Central, Floor 6, 236 Gray’s Inn Road, London WC1X 8HB, UK

## Abstract

As part of BioMed Central’s open science mission, we are pleased to announce that two of our journals have integrated with the open data repository Dryad. Authors submitting their research to either *BMC Ecology* or *BMC Evolutionary Biology* will now have the opportunity to deposit their data directly into the Dryad archive and will receive a permanent, citable link to their dataset. Although this does not affect any of our current data deposition policies at these journals, we hope to encourage a more widespread adoption of open data sharing in the fields of ecology and evolutionary biology by facilitating this process for our authors. We also take this opportunity to discuss some of the wider issues that may concern researchers when making their data openly available. Although we offer a number of positive examples from different fields of biology, we also recognise that reticence to data sharing still exists, and that change must be driven from within research communities in order to create future science that is fit for purpose in the digital age.

This editorial was published jointly in both BMC Ecology and BMC Evolutionary Biology.

## Background

This week we announce the integration of *BMC Ecology* and *BMC Evolutionary Biology* with the data repository Dryad. The hope behind this integration is not just to encourage authors to open up the data behind the articles they publish with us, but to facilitate it. Although the Dryad repository hosts research data from across all fields of science and medicine, it has been among the ecological and evolutionary biology research communities that deposition has most frequently been taken up [1]. It is for this reason that we have targeted these journals specifically, with a view to extending integration to other fields in the future.

On a practical level, what does this integration mean? If an author submits a paper to either of the aforementioned journals, they will receive an email with a one-time only link to Dryad with instructions on how to deposit their data, and how and where to cite the dataset in their paper using best practices from DataCite [2]. Once the paper is published, we at BioMed Central will notify Dryad, and they will update their records accordingly.

This does not mean we are changing the data-sharing policies of *BMC Ecology* and *BMC Evolutionary Biology*, at least for the moment. Like all journals published by BioMed Central, we strongly encourage all of our authors to archive, and make openly available, the data underlying their article. However, in the light of this update, we felt that this might also be a useful opportunity to speak to our authors about data policy more generally, in the hope of raising greater awareness of some of the major issues surrounding the debate.

The role of the publisher in data availability is something many publishers, especially open access publishers, have been discussing at least internally if not also externally. Many reading this will be familiar with the recent discussion around PLoS’s own change in policy, requiring that authors publishing with a PLoS journal make the data underlying the study publicly available (with rare exception) and to note their compliance with this in a Data Availability Statement [3,4]. At BioMed Central our policy states that “*submission of a manuscript to a BioMed Central journal implies that readily reproducible materials described in the manuscript, including all relevant raw data, will be freely available to any scientist wishing to use them for non-commercial purposes*” [5]. The idea that the data underlying a study should be available for *validation* of its conclusions is not unreasonable and is, indeed, a condition of submission for most respectable journals. It has to be.

In reality, however, any enforcement of sharing data is normally a private matter, with individual researchers contacting either individual authors or the publisher to request access. Many researchers are happy with such sharing, often welcoming the spur to collaboration it provides. However, the practicalities of tracking down data in this way are highly problematic. Private hard drives are not reliable; nor are they persistent. Similarly, researchers move, change jobs, and so on. Publicly available data housed in a repository removes the burden from the researcher to maintain and privately share his or her data. Indeed, sharing one’s data can be seen as a matter of convenience as well. It can be far less hassle to deposit your data immediately, only having to remember your name and the repository you put it in. In addition, “behind closed doors” sharing creates an inequality, as Poisot, Mounce, and Gravel note: “…those with good contacts have access to datasets, while others are left out” [6].

Yet, making data publicly available is very inconsistent across fields and researchers. Indeed, even some strong open access advocates have at least questioned data sharing as a policy. Proprietary and clinical data aside, why is this the case?

## Opportunity cost

Dr Erin McKiernan, a neurophysiologist working in Mexico and a strong open access advocate, points to a lack of funding for developing-world researchers and the practical implications of sharing data at the time of first publication, when that data is needed to sustain that lab through the publication of papers for 3 to 5 years to come [7]. Of course, being scooped is a huge fear of researchers, but what about the lost opportunity cost of increased collaboration or the extra data now made available to researchers in the developing world due to greater data sharing? Indeed, Dr McKiernan does recognise in her comments the possible benefit of supplementing her own data with other types of data: “…open electrophysiological or epidemiological data would certainly help me to improve the models I use in my work. I can also think of examples in which a lab could extend or support their smaller primary data set with open data.”

Ecological and evolutionary science has a long history of conducting research in less developed countries, partly because these areas of the world also happen to harbour its richest biological diversity [8]. Researchers from developed economies conducting research in these parts of the world will no doubt recognise the difficulties that their collaborators face in accessing the full gamut of resources needed to conduct quality research—from basic equipment to access to literature. The same is also true of data. Like the Declaration of Helsinki [9] in medical research, which states that research should benefit the populations on which it is conducted, many biologists are now recognising that a basic prerequisite of acquiring data from emerging economies should be that it is accessible to researchers in those countries [10]. Similarly, where research into applied problems stands to influence policy decisions in these countries, more attention and support needs to be paid to native researchers [11].

## Shared benefits

Although the infrequency of data sharing within many research fields makes it difficult to point to examples of the benefits of data sharing on collaboration, some examples can be seen in the genomics community, which has a lengthier history of sharing data. For example, the release of this microbiome dataset [12] resulted in a collaboration with the Agency for Science Technology and Research (A*STAR), who are currently using this data to build a new generation of tools for microbiome data. The publication of these tools will help to make this dataset the gold standard and reference for microbiome data, thus highlighting the authors’ research.

In 2005 in an article published by *Genome Biology*, authors using the Trace Archive—a repository for raw, unanalyzed genomic sequencing data—discovered three new species of the bacterial endosymbiont *Wolbachia pipientis* in three different species of fruit fly: *Drosophila ananassae*, *D. simulans*, and *D. mojavensis*[13]*.* The study shined a light on the benefits to researchers of having publicly available raw data.

A final star example demonstrating the benefits to collaboration and the increased pace of science when we share comes from the 2011 *E. coli* 0104:H4 outbreak in Europe, to which over 3,500 people fell ill (resulting in 53 deaths). What marks this story as particularly inspiring is its break from the usual scientific procedure of data production, data analysis, and then publication after a long process of peer-review. Due to the severity of the outbreak, the Beijing Genomics Institute (BGI) immediately released the full genome sequence of the strain within 5 days of receiving the genomic data of the outbreak sample. News of the release of the full genome sequence data was then aired via Twitter. Within 24 hours a GitHub repository had been created and further analyses were subsequently crowdsourced [14]. Within a couple of days, a potential ancestral strain had been found. Such rapid genomic analyses allowed for the origins and nature of the pathogen to be much better understood [15]. The story also exemplifies a crucial point to be made regarding scientific credit and etiquette, and the sharing of not only data but sharing the *analysis of that data*.

The open source analysis for the outbreak was published in the *New England Journal of Medicine*[16], proving that faster data dissemination and analysis through *sharing* need not have to undermine traditional scientific structures of credit.

## Blood, sweat and tears

Of course, the genomics community, with its longer history of data sharing, has some strong positive examples where data sharing has benefitted researchers, but sequencing data can differ greatly from species abundance or behavioural trait data, for example. A key point to make of the genomics community, especially with the deposition of raw data, is that these data might not be as “hard won” as datasets in other fields. Indeed, it is not unheard of that certain genomics institutes produce so much data they won’t possibly ever be able to write up papers for all of it.

An ecological dataset can last a researcher many years and many papers. A question we also must ask then is how will the *amount of and type of data* produced change when researchers are guaranteed only one paper from that dataset? Will there be an incentive to collect those more “hard won” datasets?

Many ecologists will be all too familiar with “pouring their blood, sweat, and tears” into a dataset, perhaps having gathered the data through years of field work, possibly having developed and maintained unique field sites themselves (e.g., establishing nest boxes to encourage birds to remain at their field sites, or long-term monitoring of plant communities under different experimental treatments).

For such datasets it is important to note a few things. First, one can deposit (and thus gain credit) for a dataset at early stages. One could release small, perhaps yearly, versions of the dataset, accruing many individual research products over the course of the experiment’s lifespan. Indeed, one’s research products are only ever versions of the entire story of one’s work. Will these be as useful as a dataset collected over 30 years? Probably not. But they will reflect your productivity as a scientist. And when you do publish your dataset collected over 30 years, yes, someone could use it—as you could use someone else’s, allowing you to compare and contrast an unlimited resource of data.

## A bigger picture

Consider the papers that could emerge by combining your dataset with datasets that were previously inaccessible. The emergence of new fields such as macroecology, dedicated to the analysis of large-scale multispecies datasets, relies on the availability of disparate sources of data in order to uncover broad patterns in ecological and evolutionary processes. Integrating these data across different scales of space and time is certainly a challenge—but so too is getting access to this data in the first instance [17]. Only by creating stronger community standards for access to, and annotation of, this data will higher-quality analysis by achievable in the future.

Some might argue collaboration will decrease. Why would you be contacted if your data is already out there, free to reuse? In the genomics community, collaboration has come from unintended reuses of that data. The microbiome data previously mentioned is one such example.

There is no reason why the same cannot be true among ecologists. What is needed, however, are clearer guidelines in terms of communication and etiquette on what is expected of researchers who choose to reuse data, and it is important to note that the positive examples of reuse that have been mentioned here involved proper communication with, and recognition of, the original data producer [18]. It is understandable that many researchers will be apprehensive about what could be perceived as a loss of control over their data. However, like many biologists now recognise, the benefits that data archiving can bring to the field offset many of these perceived fears, and may bring about new collaborative opportunities.

## Data management

Some of the more prominent data sharing communities, like genomics, also have a fairly standardised way of presenting data. Ecological and evolutionary data is typically very difficult to standardise, since it can be highly heterogeneous. The diversity of sub-fields collecting data on very different scales of grain, extent, and time—from marine microbes to whole terrestrial ecosystems—make these highly challenging disciplines to integrate. This is not to say it cannot be done, or that it shouldn’t be done, but rather to indicate that many fields are starting in a very different place than the genomics community.

A recent view into the future of biodiversity research puts open data at the top of a list of priorities facing the “grand challenge” of making sense of the current ecological data deluge, but recognises that much improved infrastructure and standardisation is needed to meet this challenge [19]. A key component of this will be better encoding and structuring of different forms of data through the use of controlled vocabularies and ontologies to ensure data are machine-readable and human-understandable. Many barriers still exist to the implementation of the recommendations, but the infrastructure for allowing it to happen is emerging [20].

Better data management will be essential, and will need to be written into grant applications and recognised by funders. A partner organisation of BioMed Central’s making much headway in this area are the open source metadata tracking ISA Tools [21]. These tools can be applied across the life sciences to help better describe rich metadata, making your dataset and study more reusable and reproducible. These tools are more appropriate for some studies than others, but they continue to be built on and represent a good starting point. This is not to say that more refined data management won’t mean more work at least in the short term. But perhaps data management is a skill required of a 21st century scientist. As ecologist Edmund Hart concludes in his blog on the subject, “I think we just need to own up to the fact [that] being a scientist these days requires new skills… In the 1990’s how many ecologists could do a mixed-effects model? Now I see them all the time. In the 21st century to do science better, we need more than spreadsheets with a few rows, we need to implement best practice for data management” [22].

However, even when a relatively standardised approach to data formatting and archiving exists, there can still be problems in ensuring data is properly archived and deposited in a way in which other users may easily re-use it. The phylogenetic tree repository TreeBase is the most widely used archive for this type of data, and has been known to the evolutionary biology community for many years, with many journals in the field stating deposition of data here as a requirement for publication. Yet even with this community-wide adoption, a recent analysis of the literature in this field found that only a small fraction of data was made publicly available by authors [23]. Even among those datasets that were made available, inconsistencies in formatting and labelling mean that this fraction is reduced further such that only a tiny amount of usable data is truly available even when the right technical infrastructure is in place.

The situation may be even worse in ecology, where datasets are typically much more variable and few dedicated repositories exist. Estimates of discoverability among the ecological literature are even more stark than in evolutionary biology, with perhaps as little as 1% being accessible after publication [24].

## Credit where credit is due

In terms of benefits to authors, many point to the now extra citeable research product you have in the form of a dataset. Some have mentioned a citation to a dataset “isn’t much credit at all” [25]—pointing to perhaps the disappointing truth that among funders and universities, papers still are the highest form of “productivity”. Although organisations like Mozilla Science Lab are working to counteract this [26] with organisations like GitHub, as things stand, until all funders and universities truly begin to value *all* research objects, this hierarchy will remain in place.

In addition to strong data citation guidelines, one answer we see at BioMed Central is to help researchers get credit for their data and encourage its reuse (and thus future citation) through more traditional lines of credit, such as the article. Data notes as an article type are available in many of our journals, like *GigaScience* and *BMC Research Notes*. A data note focuses on the data (the methodology behind it, its validation, its reuse potential) rather than the conclusions found after analysing it. It also offers a chance for a dataset *to be peer-reviewed*. In this way, an author can validate his or her study by shining a light on the validity, and strong reuse potential, of the data behind the study. Recognising the potential of this will, of course, require a shift in the perception of what constitutes a valuable contribution to scientific output, and a shift in the role that scientific publishing can play in ensuring that the heterogeneous data of ecology and evolutionary biology are fit for purpose in the digital age [27].

Making data publicly available is also another way for authors to add an additional research output, and of course, datasets are now recognised by the National Science Foundation [28] and other funders as a research product to be included in grant proposals. Studies have also shown that publicly available data connected to an article is associated with an increased citation rate [29-31]. Indeed, Piwowar and Vision recently found the increased rate can be as much as 30%, depending on the length of time the data has been public [32]. Publicly available data greatly points to increased research impact for individual researchers.

## Transparency and trust

Another incentive behind sharing data is, of course, the validation of research. In November 2009 a hacker entered the computer system at the Climate Research Unit at the University of East Anglia and exposed emails and documents showing climate scientists not only distorting data to exacerbate evidence of global warming but refusing to share raw data with critics of their work. In February 2010 a poll found a 30% drop in the past year in the percentage of British adults believing in climate change [33]. Incidences like “Climategate” are not only damaging to the reputation of all scientists but also are a detriment to public understanding of science, which is often the evidence base for important policy decisions, such as in the case of climate change.

The open availability of data ensures transparency and traceability of results, which may be checked by anyone wishing to do so. For ecological science, this is especially important since researchers working on field-based studies may have far greater difficulty in replicating experiments under differing environmental conditions than would be the case under a controlled laboratory environment [24]. Development of standardised metadata to trace provenance, especially for studies integrating many disparate data sources, will be crucial in ensuring future science meets the highest standards of quality and reproducibility.

## Concluding remarks

We are now in an age where communication across geographic and cultural barriers has been facilitated like never before, and there is little excuse for why the adoption of community standards for better data management cannot be achieved. There is also little excuse for continuing with the current loss in ecological and evolutionary data that has preceded the digital age. Think of the value that access to the past century of ecological and evolutionary literature would have to researchers working today, the many thousands of labour-hours expended collecting biological knowledge across many scales of time and space. Preventing the loss of this knowledge for future generations of biologists depends on the decisions of the research community—and it’s never been easier than now.

Stories, positive and negative, point to the benefits of data sharing, but we won’t know all the benefits, nor exactly how data sharing will change the way researchers practice science, until sharing data becomes standard. Meanwhile, as a publisher we’re in a difficult position. On the one hand, as an open access publisher, a major drive behind nearly everything we do is to make publishing research easy and painless. Publishing is a service to an author. On the other hand, we are driven by an open science mission that we believe not only makes better science but a better world. We are still discussing internally what this means for our data sharing policy, but in the meantime we are excited to see the recent discussion around open data taking place and encourage our authors to voice their own thoughts on the matter in the comments below.

It seems likely that meeting the challenges facing the natural world in the Anthropocene era will require large-scale global collaboration among researchers across ecology and evolutionary biology. We hope that the long-term benefits of opening up access to data for everyone are likely to outweigh some of the shorter-term difficulties of data sharing, and strongly encourage all of our authors to make their data openly available. We’re ready to work with researchers to ensure that facilitating this is made possible across the board, and pleased to endorse new initiatives, like our integration with Dryad, that seek to make this happen.

## Competing interests

AK, SH and CF are employees at BioMed Central.

## Authors’ contributions

AK wrote the first draft and SH and CF contributed additional edits to the text. All authors read and approved the final manuscript.

## References

[B1] Dryad Integrated Journals List http://datadryad.org/pages/integratedJournals

[B2] DataCite: Why Cite Data? http://www.datacite.org/whycitedata

[B3] PLoS Data Sharing Policyhttp://www.plosone.org/static/policies#sharing

[B4] Bloom T Ganley E Winker M Data access for the open access literature: PLOS’s data policy PLoS Biol 2014 12 2 e1001797 doi:10.1371/journal.pbio.1001797 10.1371/journal.pbio.1001797

[B5] BioMed Central Editorial Policies: Supporting Datahttp://www.biomedcentral.com/about/supportingdata

[B6] Poisot TE Mounce R Gravel D Moving toward a sustainable ecological science: don’t let data go to waste! Ideas Ecol Evol 2013 6 2 11 19 http://library.queensu.ca/ojs/index.php/IEE/article/view/4632

[B7] My concerns about PLOS’s new open data policyhttp://emckiernan.wordpress.com/2014/02/26/my-concerns-about-ploss-new-open-data-policy/

[B8] Myers N Mittermeier RA Mittermeier CG da Fonseca GAB Kent J Biodiversity hotspots for conservation priorities Nature 403 853 858 http://www.nature.com/nature/journal/v403/n6772/abs/403853a0.html 10706275 10.1038/35002501

[B9] WMA Declaration of Helsinki - Ethical Principles for Medical Research Involving Human Subjectshttp://www.wma.net/en/30publications/10policies/b3/19886379

[B10] Drew AJ Buxman CL Holmes DD Mandecki DL Mungkaje AJ Richardson AC Westneat MW Biodiversity inventories and conservation of the marine fishes of Bootless Bay, Papua New Guinea BMC Ecol 2012 http://www.biomedcentral.com/1472-6785/12/15 10.1186/1472-6785-12-15 PMC3515464 22849436

[B11] Milner-Gulland EJ Barlow J Cadotte MW Hulme PE Kerby G Whittingham MJ Ensuring applied ecology has impact J Appl Ecol 2012 49 1 5 http://onlinelibrary.wiley.com/doi/10.1111/j.1365-2664.2011.02102.x/pdf 10.1111/j.1365-2664.2011.02102.x

[B12] Li S Guan Y Zhang W Zhang F Cai Z Wu W Zhang D Jie Z Liang S Shen D Qin Y Xu R Wang M Gong M Yu J Zhang Y Han L Lu D Wu P Dai Y Sun X Li Z Tang A Zhong S Li X Chen W Zhang M Zhang Z Chen H Qin J Li Y Wang J Type 2 Diabetes gut metagenome (microbiome) data from 368 Chinese samples and updated metagenome gene catalog GigaSciencehttp://dx.doi.org/10.5524/100036

[B13] Salzberg SL Dunning Hotopp JC Delcher AL Pop M Smith DR Eisen MB Nelson WC Serendipitous discovery of Wolbachia genomes in multiple Drosophila species Genome Biol 2005 6 R23 http://genomebiology.com/2005/6/3/r23 10.1186/gb-2005-6-3-r23 15774024 PMC1088942

[B14] E. coli O104:H4 Genome Analysis Crowdsourcing https://github.com/ehec-outbreak-crowdsourced/BGI-data-analysis/wiki

[B15] I Don’t Think the German Outbreak E. coli Strain Is Novel: Something Very Similar Was Isolated Ten Years Agohttp://scienceblogs.com/mikethemadbiologist/2011/06/03/i-dont-think-the-german-e-coli/

[B16] Rohde H Qin J Cui Y Li D Loman NJ Hentschke M Chen W Pu F Peng Y Li J Xi F Li S Li Y Zhang Z Yang X Zhao M Wang P Guan Y Cen Z Zhao X Christner M Kobbe R Loos S Oh J Yang L Danchin A Gao GF Song Y Li Y Yang H The E. coli O104:H4 Genome Analysis Crowd-Sourcing Consortium Open-Source Genomic Analysis of Shiga-Toxin–Producing E. coli O104:H4 New Eng J Med 2011 365 718 724 http://www.nejm.org/doi/full/10.1056/NEJMoa1107643 10.1056/NEJMoa1107643 21793736

[B17] Beck J Ballesteros-Mejia L Buchmann CM Dengler J Fritz SA Gruber B Hof C Jansen F Knapp S Kreft H Schneider AK Winter M Dormann CF What’s on the horizon for macroecology? Ecography 2012 35 8 673 683 http://onlinelibrary.wiley.com/doi/10.1111/j.1600-0587.2012.07364.x/abstract 10.1111/j.1600-0587.2012.07364.x

[B18] Roche DG Lanfear R Binning SA Haff TM Schwanz LE Cain KE Kokko H Jennions MD Kruuk LEB Troubleshooting public data archiving: suggestions to increase participation PLoS Biol 2013 http://www.plosbiology.org/article/info%3Adoi%2F10.1371%2Fjournal.pbio.1001779 10.1371/journal.pbio.1001779 PMC3904821 24492920

[B19] Hardisty A Roberts D The Biodiversity Informatics Community A decadal view of biodiversity informatics: challenges and priorities BMC Ecol 2013 13 16 http://www.biomedcentral.com/1472-6785/13/16 10.1186/1472-6785-13-16 23587026 PMC3843378

[B20] Moritz T Krishnan S Roberts D Ingwersen P Agosti D Penev L Cockerill M Chavan V Towards mainstreaming of biodiversity data publishing: recommendations of the GBIF Data Publishing Framework Task Group BMC Bioinforma 2011 12 Supp 15 S1 http://www.biomedcentral.com/1471-2105/12/S15/S1 10.1186/1471-2105-12-S15-S1 PMC3287444 22373150

[B21] ISA toolshttp://www.isa-tools.org/

[B22] Just Get Over Yourself and Share Your Datahttp://emhart.info/blog/2014/03/04/data-sharing/

[B23] Stoltzfus A O’Meara B Whitacre J Mounce R Gillespie EL Kumar S Rosauer DS Vos RA Sharing and re-use of phylogenetic trees (and associated data) to facilitate synthesis BMC Research Notes 2012 http://www.biomedcentral.com/1756-0500/5/574 10.1186/1756-0500-5-574 PMC3583491 23088596

[B24] Reichman OJ Jones MB Schildhauer MP Challenges and opportunities of open data in ecology Science 2011 331 6018 703 705 http://www.sciencemag.org/content/331/6018/703.short 10.1126/science.1197962 21311007

[B25] I own my data, until I don’thttp://smallpondscience.com/2014/03/03/i-own-my-data-until-i-dont/

[B26] Code as a research object: a new projecthttp://mozillascience.org/code-as-a-research-object-a-new-project/

[B27] Chavan V Penev L The data paper: a mechanism to incentivize data publishing in biodiversity science BMC Bioinforma 2011 12 Suppl 15 S2 http://www.biomedcentral.com/1471-2105/12/S15/S2 10.1186/1471-2105-12-S15-S2 PMC3287445 22373175

[B28] National Science Foundation Grant Proposal Guide. Chapter II - Proposal Preparation Instructions http://www.nsf.gov/pubs/policydocs/pappguide/nsf13001/gpg_2.jsp#IIC2fic

[B29] Gleditsch NP Metelits C Strand H Posting your data: will you be scooped or will you be famous? Int Stud Perspect 2003 4 1 89 97 http://www.prio.no/Publications/Publication/?x=600

[B30] Piwowar HA Day RS Fridsma DB Sharing detailed research data is associated with increased citation rate PLoS ONE 2007 2 3 e308 doi:10.1371/journal.pone.0000308 10.1371/journal.pone.0000308 17375194 PMC1817752

[B31] Pienta AM Alter GC Lyle JA The enduring value of social science research: the use and reuse of primary research data Paper presented at “The Organisation, Economics and Policy of Scientific Research” workshop, Torino, Italy, in April, 2010 http://deepblue.lib.umich.edu/handle/2027.42/78307

[B32] Piwowar HA Vision TJ Data reuse and the open data citation advantage Peer J 2013 1 e175 https://peerj.com/articles/175/24109559 10.7717/peerj.175 PMC3792178

[B33] Jowit J Sharp decline in public’s belief in climate threat, British poll reveals Guardian 2010 9 http://www.theguardian.com/environment/2010/feb/23/british-public-belief-climate-poll

